# CD90(+) Mesothelial-Like Cells in Peritoneal Fluid Promote Peritoneal Metastasis by Forming a Tumor Permissive Microenvironment

**DOI:** 10.1371/journal.pone.0086516

**Published:** 2014-01-21

**Authors:** Joji Kitayama, Shigenobu Emoto, Hironori Yamaguchi, Hironori Ishigami, Toshiaki Watanabe

**Affiliations:** Department of Surgical Oncology, University of Tokyo, Tokyo, Japan; Cedars-Sinai Medical Center, United States of America

## Abstract

The peritoneal cavity is a common target of metastatic gastrointestinal and ovarian cancer cells, but the mechanisms leading to peritoneal metastasis have not been fully elucidated. In this study, we examined the roles of cells in peritoneal fluids on the development of peritoneal metastasis. We found that a minor subset of human intraperitoneal cells with CD90(+)/CD45(−) phenotype vigorously grew in culture with mesothelial-like appearance. The mesothelial-like cells (MLC) displayed the characteristics of mesenchymal stem cell, such as differentiating into adipocytes, osteocytes, and chondrocytes, and suppressing T cell proliferation. These cells highly expressed type I collagen, vimentin, α-smooth muscle actin and fibroblast activated protein-α by the stimulation with TGF-β, which is characteristic of activated myofibroblasts. Intraperitoneal co-injection of MLCs with the human gastric cancer cell line, MKN45, significantly enhanced the rate of metastatic formation in the peritoneum of nude mice. Histological examination revealed that many MLCs were engrafted in metastatic nodules and were mainly located at the fibrous area. Dasatinib, a potent tyrosine kinase inhibitor, strongly inhibited the proliferation of MLCs but not MKN45 *in vitro*. Nevertheless, oral administration of Dasatinib significantly inhibited the development of peritoneal metastasis of MKN45, and resulted in reduced fibrillar formation of metastatic nodules. These results suggest floating MLCs in the peritoneal fluids support the development of peritoneal metastasis possibly through the production of the permissive microenvironment, and thus the functional blockade of MLCs is a reasonable strategy to treat recurrent abdominal malignancies.

## Introduction

Peritoneal metastases frequently occur in recurrent abdominal malignancies, such as stomach and ovarian cancers. Peritoneal cancer recurrence is likely mediated by intraperitoneal free tumor cells, which are expoiliated from the serosal surface of primary tumors [1], [2]. The peritoneal cavity is the largest free space in the human body, contains a large amount of adiposed tissue and is covered by a mesothelium, which has a smooth and nonadhesive surface that facilitates intracoelomic movement. In addition to its unique anatomical structure, the peritoneal cavity contains many types of immune cells, such as lymphocytes, macrophages, and granulocytes, and mesothelial cells, which contribute to direct cell-cell contacts between tumor cells. Since the microenvironment is essential for regulating tumor development and metastasis [3], [4], the anatomical and physiological characteristics of the peritoneal cavity are considered to play critical roles in the development and progression of peritoneal metastasis.

Mesothelial cells form a monolayer lining around the entire surface of the abdominal cavity and intraabdominal organs. In addition to secreting large amounts of lubricant to prevent friction between serosal surfaces, mesothelial cells are thought to contribute to fluid transport, coagulation, fibrinolysis and antigen presentation [5]. Although mesothelial cells were originally cultured from omental tissues, cells with similar morphology have been reported to arise from malignant ascites. It was initially reported, over 30 years ago, that *in vitro* cultures of malignant effusions develop large pleomorphic cells with clear ovoid nuclei and mesothelial characteristics [6], [7]. Similar cell types were obtained from the effluent fluids of patients with chronic renal failure who underwent continuous ambulatory peritoneal dialysis [8]–[11]. Moreover, these cells were found to be incorporated into peritoneal wound surfaces and contribute to the regeneration of the mesothelium [12]. These observations suggest that mesothelial cells or their progenitors exist as free-floating cells in abdominal cavity to repair the mesothelial lining in case of peritoneal injury.

In this study, we examined intraperitoneal free cells from ascites or peritoneal lavages from patients with gastrointestinal cancer. We found that CD90(+)/CD45(−) cells comprise a minor subpopulation of floating intraperitoneal cells. However, culturing these cells *in vitro* revealed their vigorous growth rate and morphology which was identical to mesothelial cells. Interestingly, these cells also had the characteristics of mesenchymal stem cells (MSC) owing to their differentiation potential and immunosuppressive capacity. Accordingly, we classified CD90(+)/CD45(−) cells as mesothelial-like cells (MLC), and investigate their contribution to the development of peritoneal metastasis. Finally, we tested the thearpeutic potential of the functional inhibition of MLC against peritoneal metastasis.

## Materials and Methods

### Monoclonal Antibodies and Reagents

All the informations on mAbs used in this study was summarized in Table 1. In addition, Fc-blocker and 7-Amino-ActinomycinD(7-AAD)to stain dead cells were purchased from Becton-Dickinson (San Jose, CA). PKH26 were from Sigma-Aldrich (St. Louis, MO). The mesenchymal stem cell differentiation kit was obtained from R&D (Minneapolis, MN). Oil red, Alizarin red, and Truisine blue were from Sigma-Aldrich (St. Louis, MO). Carboxyfluorescein diacetate succinimidyl ester (CSFE) was purchased from Cayman (Ann Arbor, MI) and anti-CD3 mAb was purchased from Imgenex (SanDiego, CA). Imatinib and Dasatinib were purchased from Cell Signaling Technology (Danvers, MA).

**Table 1:** Summary of antibodies used in this study.

Antigen	Species/isotype	Clone name	Fluorophore	Company
Hematopoetic marker				
CD9	mouse IgG1	M-L13	FITC	Becton Dickinson
CD10	mouse IgG1	HI10a	FITC	Becton Dickinson
CD11b	mouse IgG1	ICRF44	PE	Becton Dickinson
CD34	mouse IgG1	563	PE	Becton Dickinson
CD45	mouse IgG1	HI30	FITC, perCP	Becton Dickinson
HLA-DR	mouse IgG2a	G46-6	PE	Becton Dickinson
Epithelial cell marker				
Cytokeratin	mouse IgG2a	CAM5.2	FITC	Becton Dickinson
CD326	mouse IgG1	HEA-125	PE	Miltenyi
Mesenchymal (fibroblast) marker				
Vimentin	mouse IgG1	VI-RE/1	(−)	Abcam
α-smoth muscle actin	mouse IgG2a	1A4	(−)	R&D
Type I collagen	rabbit Ig (poly)		(−)	Chemicon
Fibroblast activated protein-α	Rat IgG2a	D8	(−)	Vitatex
Mesothelial cell marker				
Calretinin	mouse IgG1	DAK Calret 1	(−)	DAKO
HBME	mouse IgM	HBME	(−)	Abcam
Mesothelin	rabbit IgG	EPR4509	(−)	Abcam
Mesenchymal stem cell marker				
CD29	mouse IgG1	4B4	FITC	Beckman Coulter
CD44	mouseIgG2b	G44-26	FITC	Becton Dickinson
CD73	mouse IgG1	AD2	FITC	Miltenyi
CD90	mouse IgG1	DG3	FITC,PE	Miltenyi
CD105	mouse IgG1	266	FITC	Becton Dickinson
CD166	mouse IgG1	3A6	PE	Beckman Coulter
Differentiation marker				
FABP-4	Goat polyclonal		(−)	R&D
Osteocalcin	mouse IgG		(−)	R&D
Aggrecan	Goat polyclonal		(−)	R&D
Isotype Control Anitibody				
mouse IgG1		APC-Cy7	(−), FITC	Becton Dickinson
mouse IgG1		APC-H7	PE	Becton Dickinson
mouse IgG2a		MOPC-21	FITC	Becton Dickinson
mouseIgG2b		27–35	FITC	Becton Dickinson
mouse IgM		MM30	(−)	Abcam
rat IgG		polyclonal	(−)	Abcam
rabbit IgG		polyclonal	(−)	R&D
Goat IgG		polyclonal		R&D
Secondary antibodies				
anti-mouse IgG	Goat F(ab')2		FITC, PE	Becton Dickinson
anti-mouse IgM	Goat F(ab')2		FITC	Invitrogen
anti-rat IgG	Goat F(ab')2		FITC, PE	Santa Cruz
anti-rabbit IgG	Goat F(ab')2		FITC	Becton Dickinson

### Cell Culture

This study was carried out in accordance with the Declaration of Helsinki and was approved by the Institutional Review Board of the University of Tokyo (Permit No:10034). The written informed consent was obtained from each patient. Intraperitoneal free cells were obtained from peritoneal lavages or ascites recovered from patients who underwent abdominal surgery for gastric cancer or paracentesis. Informed written consent was obtained from all patients. After the centrifugation at 1500 rpm for 15 min, the pellets were resuspended in PBS+0.02% EDTA and overlaid on Ficoll-Hypaque solution (Pharmacia Biotech, Piscataway, NJ). After centrifugation at 3000 rpm for 10 min, the intermediate layer was taken and washed twice. These cells were cultured with DMEM media in Type I collagen-coated plates or flasks (IWAKI, Tokyo JAPAN). After reaching confluence, the cells were removed by treatment with 0.02% EDTA and trypsin, and passaged and cultured for up to 3 weeks.

The human gastric cancer cell line MKN45 was obtained from Riken (Tukuba JAPAN) [13], and maintained in Dulbecco’s Modified Eagle Medium (DMEM) supplemented with 10% fetal bovine serum (FBS) (Sigma, St. Louis, MO), 100 units/ml penicillin and 100 mg/ml streptomycin (Life Technologies, Inc., Grand Island, NY).

### Flow Cytometry

For immunostaining, 1**×**10^6^ cells were incubated with 10 µl of Fc-blocker for 20 min and then incubated with FITC or PE-conjugated mAbs for 30 min in 4°C as per the manufacturer’s recommendation. In the case of indirect staining, cells were washed and incubated with anti-mouse or anti-rabbit IgG for an additional 30 min. After washing, the cells were then incubated with PE-conjugated anti-CD90 mAb. In the staining of the cultured cells, cells were fixed and permeabilized using BD Cytofix/Cytoperm (Becton-Dickinson, San Jose, CA) before staining. Then, the expression of each antigen on CD90(+) cells was analyzed using FACS-Caliber (Becton-Dickinson, San-Jose, CA).

### Fluorescence Microscopy

The peritoneal cells were cultured in 8 well slide chambers under the same culture condition for weeks. After fixation with 4% Formaldehyde for 30 min, the cells were stained with FITC-conjugated anti-CD45 and PE-conjugated antiCD90 mAbs for 30 min and observed with a fluorescence stereomicroscope (BZ8000, Keyence, Osaka, Japan). For intracytoplasmic staining of Cytokeratin, Vimentin and FAP-α, the cells were permeablized by 0.02% triton-X after fixation, incubated with each mAb, and incubated with PE-conjugated secondary Abs.

### Differentiation Assay

The peritoneal cells were cultured for 1 week and transferred to 8 well slide chambers and cultured with adipogenic, osteogenic or chondrogenic media media for additional 7∼18 days, and then fixed with 4% Formaldehyde for 30 min. For detection of lipid droplets, the samples were washed with 2-propanol and then incubated with 60% Oil red for 15 min. For the detection of bone or cartilage substrates, the fixed samples were incubated with 1% Alizalin Red or 0.05% Truisine Blue for 30 min, respectively. In addition, the samples were immunostained with specific Abs to FABP4, Osteocalcin or Aggrecan, followed by FITC conjugated secondary Abs, using mesenchymal stem cell differentiation kit (R&D) according to manufacturer’s instruction.

### Proliferation Assay

MKN45 cells or cultured peritoneal cells (5×10^3^ cells in 100 µl/well) were seeded into a 96-well-microtiter plate in DMEM media in the presence or absence of Imatinib and Dasatinib. After incubation at 37°C in 5% CO_2_ for 48 hours, the number of living cells was measured using an MTS assay (Promega, Madison, WI) according to the manufacturer’s instructions.

### T cell Proliferation Assay

Under the approval by the Institutional Review Board of the University of Tokyo, peripheral blood derived mononuclear cells (PBMC) were obtained from healthy volunteers and stained with Carboxyfluorescein diacetate succinimidyl ester (CSFE) for 30 min. Twenty-four well plates were filled with 1 ml PBS containing 5 µg/ml of anti-CD3 mAb and incubated for 24 hours. After washing the wells 3 times, the stained cells (1**×**10^6^) were cultured on anti-CD3 coated plates in the presence or absence of MLC for 4 days. Then, fluorescent intensities of CFSE in CD3(+) T cells were analyzed with FACS. In some experiments, MLCs (1**×**10^5^) were added on culture inserts within the same well to inhibit the direct cell-cell contact.

### In vivo Experiments

This study protocol was carried out in strict accordance with the recommendations in the Guide for the Care and Use of Laboratory Animals of the National Institutes of Health. The protocol was approved by the Committee on the Ethics of Animal Experiments of the University of Tokyo (Permit No:12-P-85) Four-week-old specific-pathogen-free conditioned female BALB/c nude mice were purchased from Charles River Japan, Inc. (Yokohama, Japan), and maintained in a temperature controlled, light cycled room. At 5 weeks after birth, the mice were intraperitoneally (IP) inoculated with MKN45 cells (5**×**10^4^∼1**×**10^6^) suspended in 0.5 ml Hanks balanced solution (HBSS). In the case of co-transfer experiments, 5**×**10^5^ MLCs suspended in 0.5 ml HBSS were mixed just before IP inoculation. After 3 weeks, the mice were sacrificed and peritoneal metastatic nodules were counted. Then, the peritoneal nodules were excised, fixed for 1 h in 10% neutral buffered formalin and the red fluorescence of PKH26 was observed with fluorescence stereomicroscopy. For further analysis, the samples were washed overnight in PBS containing 10% sucrose at 4C, embedded in optimal cutting temperature compound (Tissue-Tek, Sakura Finetek, Torrance, CA, USA), snap frozen in dry-iced acetone, and sectioned (5 µm thickness). The sections were stained using the Masson-Trichrome method to visualize collagen fibers. For Dasatinib treatment, 1**×**10^6^ MKN45 cells and 5**×**10^5^ MLCs were co-injected into the nude mice and Dasatitib (50 mg/kg) in 1.0 ml PBS or PBS alone were orally administrated every day since 3 days after tumor inoculation. Two weeks later, the mice were sacrificed and macroscopic metastases in the peritoneum were counted. Then, the tumors were excised and examined after sectioning.

### Statistical Analysis

The results were statistically examined by paired Student’s t tests or Wilcoxons test when appropriate. Results are given as mean±SD, and differences with P<0.05 were considered to be significant.

## Results

### Peritoneal Fluids Contain a Distinct Cell Population with a CD90(+)/CD45(−)/CD326(−) Phenotype

**Figure 1:**
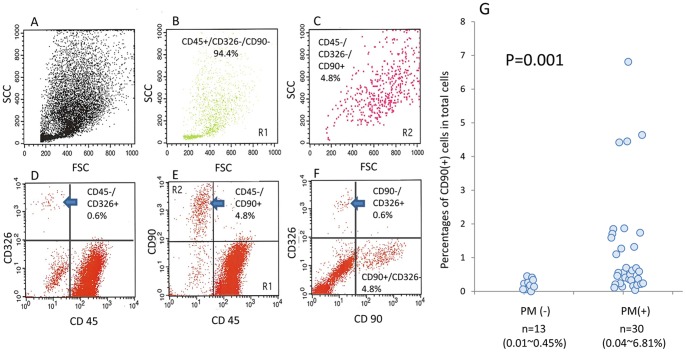
Intraperitoneal free cells were recovered from peritoneal lavages or ascites recovered at laparotomy from patients of gastrointestinal cancer and stained with FITC-conjugated anti-CD90, PE-conjugated anti-CD326 and PerCP-conjugated anti-CD45 and analyzed by FACS. FACS profiles of a representative case were shown in A∼F. A: FSC/SCC, D: PerCP/PE, E: PerCP/FITC, F: FITC/PE. B and C show the FSC/SCC profiles of the cells located in Region 1 (CD45+, CD90−) and Region 2 (CD45−, CD90+), respectively. G: The cells were immunostained with FITC-conjugated anti-CD45 and PE-conjugated anti-CD90 mAbs and the ratio of CD45(−)CD90(+) cells were calculated in patients with or without peritoneal metastasis. The percentages of each cell population were calculated against total acquired cell counts of 10^4^.

The phenotypes of cells in human peritoneal fluids were examined with flow cytometry. In all cases, the majority of the cells were CD45(+) leukocytes. In highly advanced cases with peritoneal metastasis, CD326(+)/CD45(−) tumor cells were detected (Fig. 1). Additionally, we found that a minor population of cells in peritoneal cavity were CD90(+) cells. The CD90(+) cells were detected at a relatively high FSC and SCC region as compared with CD45(+) leukocytes (Fig. 1 B,C). These cells did not express either CD45 or CD326 (Fig. 1 D–F), suggesting that they are distinct from other peritoneal cells. The percentage of CD90(+)/CD45(−)/CD326(−) cells was generally low in patients without peritoneal metastasis (0.01∼0.45%). However, in patients with peritoneal metastasis, the proportion of these cells was significantly elevated (0.04∼6.81%, p = 0.001) (Fig. 1G).

### Intraperitoneal CD90(+) are Highly Proliferative *in vitro* and have Mesenchymal Stem Cell (MSC) Characteristics

**Figure 2:**
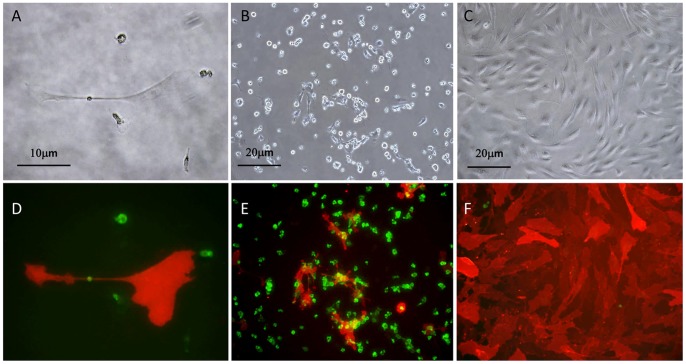
Appearance of the peritoneal cells cultured for 7(A,D), 14 days (B,E) and 20 days (C,F). A∼C: phase contrast, D∼F; photomicrograph following immunofluorescence staining. Cells were fixed and stained with FITC-conjugated anti-CD45 mAb and PE-conjugated anti-CD90 mAb, and two images to detect FITC and PE were merged in D∼F. Representative images are shown.

Cells recovered from the peritoneal cavity were maintained as bulk cultures in 10% FCS+DMEM media on type I collagen coated plates. Under these conditions, the CD90(+) cells grew vigorously in an adherent manner (Fig. 2). During several days of culture, these adherent cells were enlarged in size with variable morphology and often showed close contact with small round cells, which were CD45(+) leukocytes (Fig. 2 A,B,D,E). After culturing for 2–3 weeks, most of the adherent cells were composed of CD90(+)CD45(−) cells (Fig. 2 C,F). At this stage, the morphology of the cells was mostly fibroblastoid with some epitheloid, which are consistent with mesothelial cell characteristics as previously reported [6], [9]. When CD90(+) cells were enriched from the initial cell population by MACS method, they developed mesothelial-like cells with epitheloid appearance, while CD90(+) cell-depleted fraction never showed such vigorous growth (Fig. S1).

**Figure 3:**
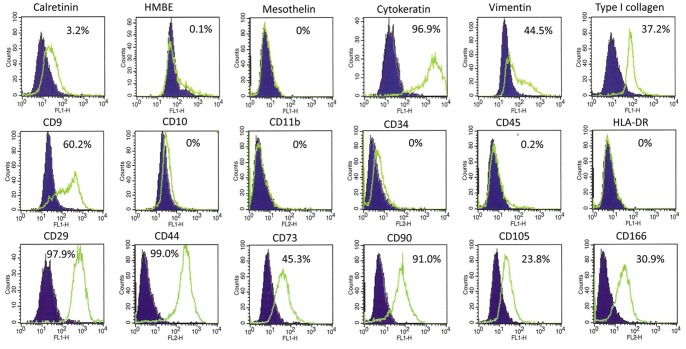
Cells recovered from a patient with peritoneal metastasis were cultured for 2 weeks and their phenotypes were examined by FACS. The cells were detached from culture plate and fixed and permeabilized using BD Cytofix/Cytoperm (Becton-Dickinson, San Jose, CA) before immunostaining. At this time point, some small hematopoietic cells contaminated the bulk culture, but were excluded by gating on FSC/SCC profile. Green lines denote the fluorescent profiles of the indicated antigens and filled lines correspond to negative controls. Each value represents the percentages of the cells with positive expression for indicated antigens in 10^4^ of gated cells.

The antigen expression patterns of cultured intraperitoneal cells were shown in Fig. 3. After culturing for more than 2 weeks, these cells strongly expressed cytokeratin and moderately expressed vimentin and type I collagen, suggesting they possessed both epithelial and fibroblastoid character. Among the mesothelial cell antigens, they slightly expressed calretinin and did not express Hector Battifora-mesothelin (HBME)-1 or mesothelin. In addition, these cells expressed mesenchymal stem cell (MSC)-related antigens such as CD29, CD44, CD73, CD105 and CD166. In contrast, with the exception of CD9, they did not express hematopoetic cell-related antigens such as CD10, CD11b, CD34, CD45, and HLA-DR.

**Figure 4:**
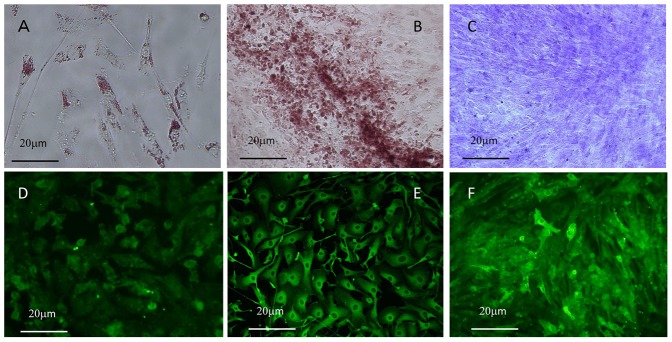
Ascitic cells derived from a patient with peritoneal metastasis were cultured for 1(A) and immunostained with anti-FABP4 Ab (D). The cells were cultured with osteogenic or chondrogenic media for 18(B) or Truisine Blue (C), respectively. Also, Osteocalcin (E) or Aggrecan (F) was immunostained using specific Abs followed by FITC conjugated secondary Abs.

The phenotypic similarity of these cells to MSCs prompted us to examine whether they have a capacity to differentiate into multiple cell types. After 1 week of culture in adipogenic differentiation media, the cells strongly expressed Oil-red stained granules (Fig. 4A) and FABP-4 antigen (Fig. 4D), highlighting the capacity of these cells to differentiate into adipocytes. Similarly, the cells expressed osteocalcin (Fig. 4E) and aggrecan (Fig. 4F) after 2∼3 weeks of culture under the optimal conditions, and showed clear staining with Alizalin red (Fig. 4B) or Trusine blue (Fig. 4C), respectively, suggesting they also have the potential to differentiate into osteocyte and chondrocyte. These results clearly show that the cells recovered from human peritoneal fluid possess MSC-like properties. Together with their morphological similarity to mesothelial cells, our observations allowed us to designate these cells as mesothelial-like cells (MLC) for the remainder of this study.

**Figure 5:**
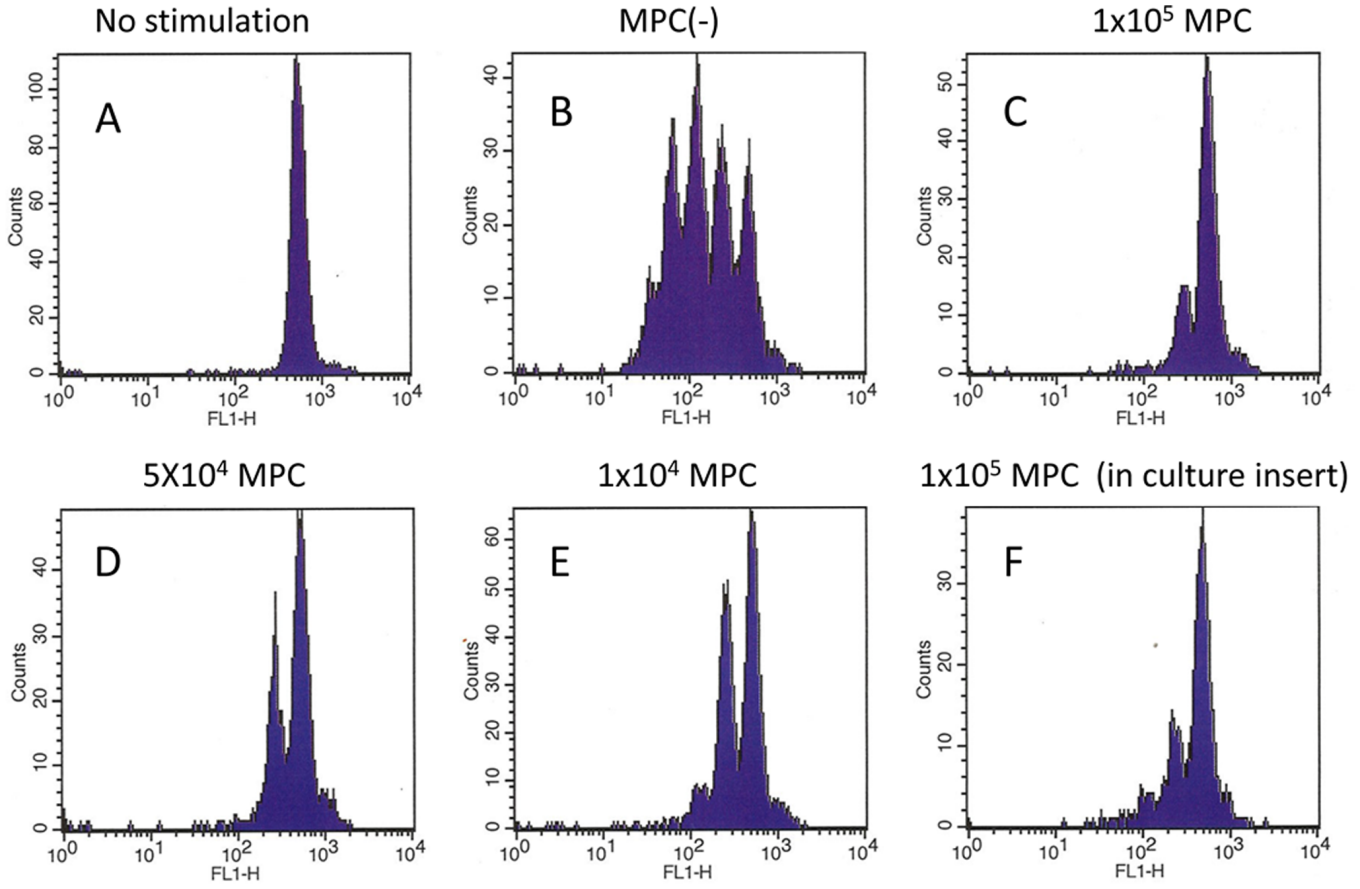
PBMC (1×10^6^) derived from healthy volunteers were stained with CFSE and cultured on plastic (A) or anti-CD3 coated (B∼F) plates in the presence or absence of the indicated number of MLCs for 4 days, and CFSE fluorescence intensities in CD3(+) T cells were analyzed with FACS. In F, MLCs (1**×**10^5^) were added on culture inserts within the same well. Data shown is representative of results from 3 different experiments.

Since the immunosuppressive activity of MSCs is well known, we next examined whether the presence of the MLC inhibits proliferation of T cells stimulated with anti-CD3. As shown in Fig. 5A∼E, a dilution assay using CFSE clearly showed that the addition of MLCs suppressed T cell activation in a dose dependent manner. The addition of the MLCs to PBMCs at a 1∶10 ratio almost completely inhibited T cell proliferation (Fig. 5C), and a 1∶100 ratio of MPCs to PBMC yielded significant inhibition (Fig. 5E). This suppression did not require cell-cell contact, since addition of MLCs in culture separated by a double chamber was sufficient to inhibit T cell proliferation (Fig. 5F).

### Cultured MLCs Highly Express Type I Collagen and Differentiate into Myofibroblast after TGF-β Treatment

**Figure 6:**
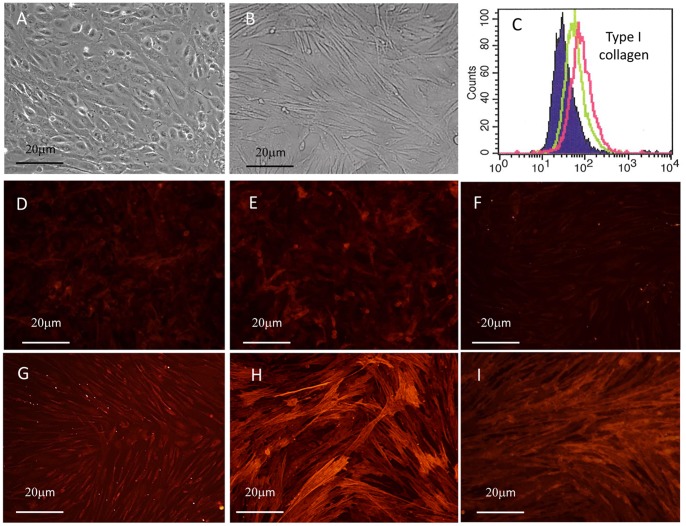
Phase contrast images of ascitic cells derived from a patient with peritoneal metastasis were cultured with (B) or without (A) 10 ng/ml TGF-β for 24 hours. C: Expression of Type I Collagen was quantitatively evaluated using FACS. MLCs treated with (Red line) or without (Green line) 10 ng/ml TGF-β for 48 hours were detached, fixed, permeabilized and stained with rabbit Ab to Type I collagen as described Material and Methods. Shaded profile shows the negative control. Same MLCs were cultured with (G,H,I) or without (D,E,F) 10 ng/ml TGF-β for 48 hours, fixed with paraformaldehyde and stained with mouse mAbs to Vimentin (D,G), α-SMA (E,H), and FAP-α (F,I). The slides were then incubated with PE-conjugated secondary Abs, and analyzed by fluorescence microscopy.

To further examine their differentiation potential, we grew MLCs in the presence of TGF-β. Interestingly, under these conditions their morphology appeared to be fibroblastic with a marked spindle shape within 24 hours of treatment (Fig. 6 A.B). Moreover, the expression of type I collagen, as evaluated by FACS, was significantly increased after 48 hour (Fig. 6C). Similarly, the expression of vimentin, α-SMA and FAP-α were also significantly upregulated (Fig. 6D∼I, Fig. S2). These results clearly indicate that MLCs differentiate into myofibroblast after stimulation with TGF-β.

### Co-injection of MLCs with MKN45 Cells Enhanced Peritoneal Metastasis in Nude Mice

We examined whether MLCs can affect the development of peritoneal metastasis using nude mice model. As shown in Table 2, intraperitoneal (IP) transfer of MKN45 cells (1**×**10^6^) resulted in the formation of peritoneal metastasis in 50% (4/8) of mice. Transfer of 1**×**10^5^ MKN45 cells did not yield metastatic nodules. In comparison, when MLCs (5**×**10^5^) were transferred together with MKN45 cells (1**×**10^6^), peritoneal metastasis developed in all (9/9) mice. Remarkably, co-transfer of the same number of MLCs with 1**×**10^5^ MKN45 cells led to peritoneal tumor formation in 75% (6/8) of mice. Metastasis developed even in 1 of 4 mice co-transferred with 5**×**10^4^ MKN45 cells. These results indicate that MLCs have supportive roles on the development of peritoneal metastasis.

**Table 2:** Co-transfer of MLC enhances peritoneal metastasis of MKN45.

No. of MKN45	No. of MLC	Tumor developed mice
1**×**10^6^	(−)	4/8
1**×**10^6^	5**×**10^5^	9/9
1**×**10^5^	(−)	0/6
1**×**10^5^	5**×**10^5^	6/8
3**×**10^4^	(−)	0/2
3**×**10^4^	5**×**10^5^	1/4

MKN45 were intraperitoneally (IP) injected into 5 week Balb/c nude mice with or without indicated number of cultured MLCs The mice was sacrificed at 3 weeks later, and the number of mice which developed peritoneal metastasis was counted.

### MLC were Engrafted in Metastatic Nodules of MKN45 on Peritoneum

**Figure 7:**
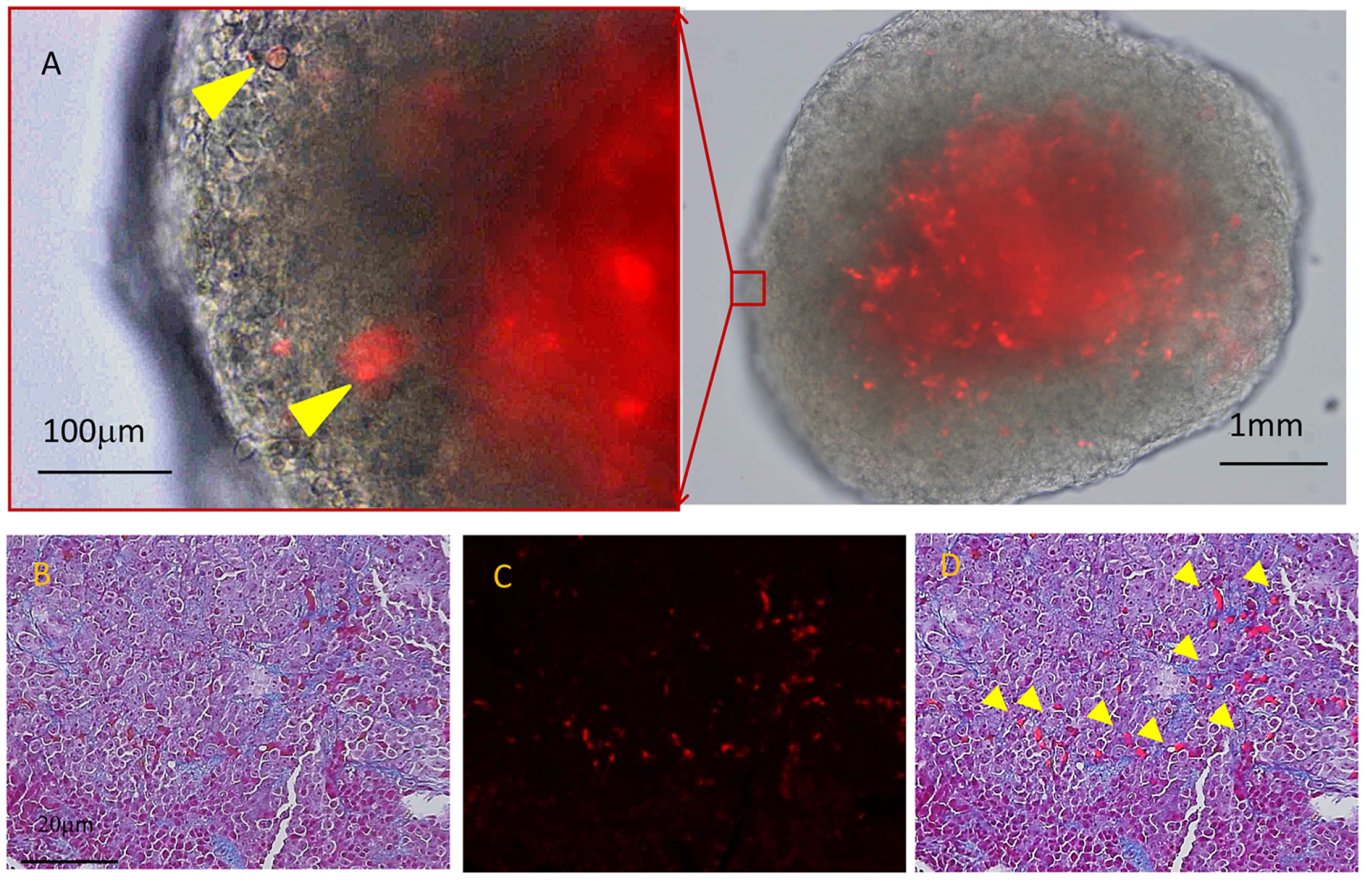
Peritoneal nodules developed in nude mouse after IP injection of MKN45 cells (1×10^6^) and PKH26-labelled MLCs (5×10^5^). A: Nodules were excised and observed under fluorescence microscope. MLCs engrafted in metastatic nodules are highlight by arrow heads. Tissue sections of the peritoneal nodules were stained using the Masson-Trichrome method and observed under light (B) and fluorescence microscopy (C), and merged (D).

To examine whether MLCs are directly incorporated in metastatic nodules, we labeled MLCs with PKH26 and IP injected them together with MKN45 cells. After 3 weeks the peritoneal nodules were excised and examined under fluorescence microscopy. As shown in Fig. 7A, many MLCs (red spots) were detected in metastatic tumors on the peritoneum. Histological analysis with Masson-Trichrome staining revealed that the PKH26-positive cells were distributed mainly at the fibrous area (Fig. 7 B∼D). Moreover, immunostaining revealed that the PKH26(+) cells were positive for FAP-α and Type I collagen (Fig. S3). These findings strongly suggest that MLCs are engrafted in metastatic nodules and actively produced collagen, which may promote the survival of MKN45 cells.

### Dasatinib Strongly Inhibits the Growth of MLCs and Suppresses the Development of Peritoneal Metastasis in vivo

**Figure 8:**
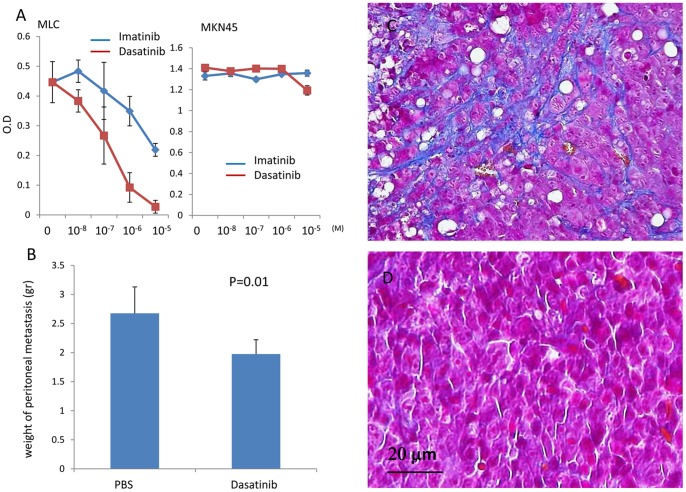
A: MLCs (1**×**10^4^) derived from ascites of a patient with peritoneal metastasis were cultured in the presence of the indicated concentrations of Imatinib or Dasatinib for 3 days. Cell proliferation was assessed by MTS assay. Data of a representative experiment from 3 different experiments are shown. B∼D: MKN45 cells (1**×**10^6^) and MLCs (5**×**10^5^) were co-injected into the peritoneum of nude mice. Dasatinib (50 mg/kg) in 1.0 ml PBS was orally administrated for 14 consecutive days starting 3 days after tumor inoculation. Two weeks later, the mice were sacrificed and macroscopic metastasis in the peritoneum were counted, excised and evaluated for the total weight (B). Tissue sections of the peritoneal nodules that developed in control (PBS) (C) and Dasatinib-treated (D) mice were stained using the Masson-Trichrome method.

To study the role of MLCs in peritoneal metastasis, we sought to identify drugs that would inhibit MLC activity. We examined many drugs which were reported to inhibit fibrotic process in previous studies [14]. Among them, Imatinib and Dasatinib significantly suppressed MLC proliferation in a dose dependent manner (Fig. 8A). In particular, Dasatinib was notably effective at a concentration of <100 nM and almost completely inhibited MLC proliferation at 10 µM. However, neither Imatinib nor Dasatinib inhibited the growth of MKN45 cells at the concentration of <10 µM.

Given that Dasatinib is an effective inhibitor of MLCs, we tested the effects of this drug on peritoneal metastasis *in vivo*. After the co-injection of MKN45 cells and MLCs, Dasatinib or PBS alone was orally administrated to mice for 14 consecutive days. As shown in Fig. 8B, total weight of peritoneal tumors was significantly reduced in mice treated with Dasatinib (1.98±0.24 g) as compared to control mice (2.68±0.45 g) (p<0.05, n = 5). Moreover, histological examination with Masson-Trichrome staining indicated that the metastatic nodules in Dasatinib-treated mice were significantly less fibrous as compared with the tumors of control mice (Fig. 8C,D). The ratio of fibrous area in peritoneal nodules was significantly reduced in Dasatinib treatment group as compared with control (0.46±0.4% vs 4.3±2.3%, p = 0.007) (Fig. S4).

## Discussion

Previous studies have shown that cells of mesothelial lineage can be cultured from peritoneal fluids such as malignant ascites [6], [7] [15] or effluents of continuous ambulatory peritoneal dialysis [8]–[11]. In those studies, mesothelial cells displayed various morphological features such as cobblestone-like, transitional, fibroblast-like spindles, as well as other features depending on culture conditions. In this study, we found that CD90(+)/CD45(−) cells exist as a minor but distinct population in peritoneal cavities of patients with gastrointestinal cancer and called them as mesothelial-like cells (MLC) by their morphological features. The MLC vigorously grew in culture and possessed a capacity to differentiate into adipocytes, osteocytes, chondrocytes and myofibroblast under appropriate culture conditions. This suggests that free mesothelial cells in the peritoneal cavity may not be fully differentiated, but rather exist as progenitors for various cell types including mesothelial cells.

In fact, cultured mesothelial cells were mesenchymal in origin but exhibited epithelial characteristics such as surface expression of microvilli, cytokeratin and tight junction, suggesting the ‘stemness’ property of mesothelial cells [16]. Moreover, Lansley et al. have recently reported that cultured mesothelial cells, isolated from omentum or peritoneal fat, can differentiate into osteoblast and adipocyte-like cells [17]. We found a similar trend for MLCs, although they were derived from peritoneal fluids and had distinct antigen expression patterns. Moreover, Ho et al. recently reported that the same CD90(+)/CD45(−) cells function as stromal progenitor cells in ascites of ovarian cancer patient [18]. These results are in line with our results, and support the concept that progenitor-like cells with a mesothelial lineage are present as free-floating cells in the peritoneal cavity [19]. Since mesothelial cells originally develop from the embryonic mesoderm, the multipotent nature of these cells may not be surprising. In fact, these observations extend the possibility that the peritoneal cavity may be a unique niche that maintains such stem cell-like cells similar to the bone marrow.

In recent years, interest in mesenchymal stem, or stromal, cells (MSC) has increased because they are useful for regenerative therapy. Although MSCs were originally isolated from hematopoetic cells in the bone marrow [20]–[22], they are also detected in various tissues and thought to contibute to tissue remodelling processes after injury or chronic inflammation [20], [23]. In fact, MSCs can now be isolated from many tissues such as adipose tissue [24], umbilical cord [25], amniotic fluid [26], dental pulp [27] and the circulatory system [28], although functional differences have been reported among the MSCs derived from various tissues [29]. Since the MLCs we identified in this study have similar differentiation potentials, surface markers, and immunosuppressive activity, we suppose that cultured mesothelial cells might be categorized as peritoneal MSCs.

Importantly, MLCs expressed high amounts of type I collagen and displayed characteristics of myofibroblasts after stimulation with TGF-β. Furthermore, IP co-injection of the cultured MLCs together with MKN45 cells resulted in enhanced tumorigenicity in nude mice, uncovering a supportive role for MLCs during cancer cell metastasis to the peritoneum. This is consistent with recent results that cultured human mesothelial cells derived from omental tissue have supportive effects that promote tumorigenesis both *in vitro* [30], [31] and *in vivo* [32]. In fact, after co-injection with MKN45 cells, many MLCs became were engrafted in metastatic nodules in the peritoneum and were detected primarily in the fibrous interstitial area of the tumor nodule. Since the addition of MLCs did not show enhanced proliferation of MKN45 cells *in vitro* (data not shown), we suppose that they may contribute to the development of peritoneal metastasis by forming favorable microenvironment for tumor growth.

Solid tumors not only consist of tumor cells but also non-malignant stromal cells and extracellular matrix (ECM), and the complex interactions among these cells and ECM critically regulates tumor progression, metastasis and chemoresistance [33], [34]. Since collagens are the most prevalent component of tumor ECM, can elicit various biochemical or biophysical functions, and act as a physical scaffold [35]–[37], active production of collagenous matrix by MLC-derived myofibroblasts in tumor sites may be a critical step in metastasis. In fact, peritoneal mesenchymal stem cells are proposed to play a major role in the pathogenesis of peritoneal fibrosing syndrome after frequent dialysis [38]. Thus, it may be possible that MLCs cause tumor related fibrosis via the same mechanisms.

Another important property of MLCs is the strong inhibitory effects on T cell proliferation through soluble factors as evidenced by our transwell culture assay. MSCs can suppress both innate and adaptive immunity making them useful for the clinical treatment of graft versus host disease (GVHD) [23], [39]. In our experiments, it is unlikely that the enhanced tumorigenicity of MKN45 cells was caused by the immunosuppressive activity co-transferred MLCs since T cell immunity is lacking in nude mice. However, MSCs have been reported to also inhibit the proliferation, cytotoxicity, and cytokine production of natural killer (NK) cells [40], [41]. Therefore, it is possible that MLC-mediate suppression of NK cells may promote metastasis, even in our xenotransplantation system. Although experiments with immunocompetent animals are necessary, the immunomodulatory effect of MLC may have a significant contribution on the development of peritoneal metastasis in human.

Rafii et al. reported that cells isolated from malignant ascites of ovarian cancer patients show novel phenotype and induce chemoresistance in tumor cells [42]. It has also been reported that stromal cells named “hospicells” inhibit T cell mediated immune response [43] and promote tumorigenicity and angiogenesis *in vivo* [44]. The MLCs used in our experiments have some functional similarities with hospicells, suggesting a possible connection in terms of cell lineage. However, hospicells express CD9 and CD10, which are not expressed in MLCs, and CD73, CD90 or CD105 are present in MPCs but lacking in hospicells. Moreover, the differentiation properties of MLCs are not similar to those in hospicells. Experiments *in vivo* showed that hospicells enhance tumorigenicity of ovarian cancer cells by increasing microvascularization. In our model, co-injection of MLCs also enhanced tumorigenicity of gastric cancer cells, but probably through a different mechanism. The reason of the discrepancy of these two cell types might be attributed to the different culture system or different cell sources between gastric and ovarian cancers.

Imatinib and Dasatinib are tyrosine kinase inhibitors (TKI) that efficiently block TGF-β and PDGF signaling and suppress fibrotic processes in various experimental models [45]–[48]. The anti-fibrotic effects of TKI are also apparent in humans, prompting their use in clinical trials for systemic sclerosis or idiopathic pulmonary fibrosis [49], [50]. Therefore, we examined the effects of these TKI on the function of MLCs. We found that Dasatinib inhibits the growth of MLCs more potently than Imatinib. Furthermore, Dasatinib did not inhibit the growth of MNK45 cells *in vitro*. Dasatinib significantly suppressed the development of peritoneal metastasis of MKN45 cells *in vivo*, and peritoneal nodules that did form were significantly less fibrotic. Our results suggest a possibility that the functional inhibition of MLCs by Dasatinib can alter the microenvironment in peritoneal cavity, resulting in the inhibition of peritoneal metastasis.

In summary, we found that progenitor cells of mesothelial lineage are present in the human peritoneal cavity and probably function in pathways responsible for regenerating the mesothelium following peritoneal damage. These cells, however, play a supportive role on the development of peritoneal metastatic possibly by providing a permissive microenvironment for metastatic tumor cells. In other words, tumor cells might co-opt the “regeneration system” of the peritoneum to develop metastatic foci. In a similar process, bone marrow-derived stem cells, which are preferentially recruited to tumor sites and wounds, have been shown to participate in the formation stroma during tumorigenisis [51]–[53]. Thus, inhibition of regenerative mechanisms in the peritoneum may be a reasonable strategy to suppress tumor metastasis. Indeed, our *in vivo* results suggest that the addition of TKI, to inhibit MLCs, during conventional chemotherapy may be a reasonable strategy to treat peritoneal metastasis.

## Supporting Information

Figure S1 **Peritoneal cells recovered from a patient were suspended with anti-CD90 mAb conjugated with microbeads and separated to CD90(+) enriched fraction and CD90(+) depleted fraction using MACS separation kit (Miltenyi Biotec, GmbH).** Then, each fraction was cultured on Type I collagen coated plate with the same culture condition for 2 weeks and observed under the phase contrast microscope. CD90(+) enriched fraction developed mesothelial-like cells with cobblestone like appearance (A), while cells of CD90(+) depleted fraction did not proliferate and maintained round shape with some cells with fibroblastoid appearance (B).
(TIF)

Figure S2 **Peritoneal nodules developed in nude mouse after IP injection of MKN45 cells (1×10^6^) and PKH26-labelled MLCs (5×10^5^) were excised, and the tissue sections were immunostained with rabbit antibody to type I collagen (B) or rat antibody to FAP-α (D) followed by the incubation with FITC-conjugated secondary antibody, and observed under fluorescence microscopy.** (A) control rabbit IgG, (C) control rat IgG. In merged pictures, PKH26 (+) cells are shown to be positive for Type I collagen (Arrows in B) and for FAP-α (Arrowhead in D).
(TIF)

Figure S3 **MLC treated with (Red line) or without (Green line) 10 ng/ml TGF-β for 48 hours were detached, fixed, permeabilized and stained with mAbs to Vimentin, α-SMA and FAP-α as described Material and Methods.** Shaded profile shows the negative control.
(TIF)

Figure S4 **MKN45 cells (1×10^6^) and MLCs (5×10^5^) were co-injected into the peritoneum of nude mice.** Dasatinib (50 mg/kg) in 1.0 ml PBS was orally administrated for 14 consecutive days starting 3 days after tumor inoculation. Two weeks later, the mice were sacrificed and macroscopic metastasis in the peritoneum were excised, and tissue sections of peritoneal nodules of control and Dasatinib-treated mice were stained using the Masson-Trichrome method, and the percentages of fibrous area in total area were calculated in randomly selected 10 areas in 5 different tissue sections using a measurement module of BZ-H1M analyzing system (Keyence, Osaka, Japan).
(TIF)

## References

[1] Koga S, Kaibara N, Iitsuka Y, Kudo H, Kimura A, et al (1984) Prognostic significance of intraperitoneal free cancer cells in gastric cancer patients. J Cancer Res Clin Oncol 108: 236–238. 6470030 10.1007/BF00402474 PMC12253066

[2] Sodek KL, Murphy KJ, Brown TJ, Ringuette MJ (2012) Cell-cell and cell-matrix dynamics in intraperitoneal cancer metastasis. Cancer Metastasis Rev 31: 397–414. 22527451 10.1007/s10555-012-9351-2 PMC3350631

[3] Mueller MM, Fusenig NE (2004) Friends or foes - bipolar effects of the tumour stroma in cancer. Nat Rev Cancer 4: 839–849. 15516957 10.1038/nrc1477

[4] Fidler IJ (2002) The organ microenvironment and cancer metastasis. Differentiation 70: 498–505. 12492492 10.1046/j.1432-0436.2002.700904.x

[5] Mutsaers SE (2004) The mesothelial cell. Int J Biochem Cell Biol 36: 9–16. 14592528 10.1016/s1357-2725(03)00242-5

[6] Cailleau R, Mackay B, Young RK, Reeves WJ Jr (1974) Tissue culture studies on pleural effusions from breast carcinoma patients. Cancer Res 34: 801–809. 4592574

[7] Whitehead RH, Hughes LE (1975) Tissue culture studies of malignant effusions. Br J Cancer 32: 512–518. 1212414 10.1038/bjc.1975.254 PMC2024775

[8] Betjes MG, Bos HJ, Krediet RT, Arisz L (1991) The mesothelial cells in CAPD effluent and their relation to peritonitis incidence. Perit Dial Int 11: 22–26. 2049418

[9] Yanez-Mo M, Lara-Pezzi E, Selgas R, Ramirez-Huesca M, Dominguez-Jimenez C, et al (2003) Peritoneal dialysis and epithelial-to-mesenchymal transition of mesothelial cells. N Engl J Med 348: 403–413. 12556543 10.1056/NEJMoa020809

[10] Fang CC, Yen CJ, Chen YM, Shyu RS, Tsai TJ, et al (2000) Pentoxifylline inhibits human peritoneal mesothelial cell growth and collagen synthesis: effects on TGF-beta. Kidney Int 57: 2626–2633. 10844633 10.1046/j.1523-1755.2000.00123.x

[11] Rampino T, Cancarini G, Gregorini M, Guallini P, Maggio M, et al (2001) Hepatocyte growth factor/scatter factor released during peritonitis is active on mesothelial cells. Am J Pathol 159: 1275–1285. 11583955 10.1016/S0002-9440(10)62514-X PMC1850499

[12] Foley-Comer AJ, Herrick SE, Al-Mishlab T, Prele CM, Laurent GJ, et al (2002) Evidence for incorporation of free-floating mesothelial cells as a mechanism of serosal healing. J Cell Sci 115: 1383–1389. 11896186 10.1242/jcs.115.7.1383

[13] Yanagidani S, Uozumi N, Ihara Y, Miyoshi E, Yamaguchi N, et al (1997) Purification and cDNA cloning of GDP-L-Fuc:N-acetyl-beta-D-glucosaminide:alpha1–6 fucosyltransferase (alpha1–6 FucT) from human gastric cancer MKN45 cells. J Biochem 121: 626–632. 9133635 10.1093/oxfordjournals.jbchem.a021631

[14] Rodriguez EF, Monaco SE, Khalbuss W, Austin RM, Pantanowitz L (2013) Abdominopelvic washings: A comprehensive review. Cytojournal 10: 7. 23858317 10.4103/1742-6413.111080 PMC3709516

[15] Singh G, Dekker A, Ladoulis CT (1978) Tissue culture of cells in serous effusions. Evaluation as an adjunct to cytology. Acta Cytol 22: 487–489. 282748

[16] Mutsaers SE (2002) Mesothelial cells: their structure, function and role in serosal repair. Respirology 7: 171–191. 12153683 10.1046/j.1440-1843.2002.00404.x

[17] Lansley SM, Searles RG, Hoi A, Thomas C, Moneta H, et al (2011) Mesothelial cell differentiation into osteoblast- and adipocyte-like cells. J Cell Mol Med 15: 2095–2105. 21070599 10.1111/j.1582-4934.2010.01212.x PMC4394220

[18] Ho CM, Chang SF, Hsiao CC, Chien TY, Shih DT (2012) Isolation and characterization of stromal progenitor cells from ascites of patients with epithelial ovarian adenocarcinoma. J Biomed Sci 19: 23. 22330345 10.1186/1423-0127-19-23 PMC3305560

[19] Herrick SE, Mutsaers SE (2004) Mesothelial progenitor cells and their potential in tissue engineering. Int J Biochem Cell Biol 36: 621–642. 15010328 10.1016/j.biocel.2003.11.002

[20] Bianco P, Robey PG, Simmons PJ (2008) Mesenchymal stem cells: revisiting history, concepts, and assays. Cell Stem Cell 2: 313–319. 18397751 10.1016/j.stem.2008.03.002 PMC2613570

[21] Pittenger MF, Mackay AM, Beck SC, Jaiswal RK, Douglas R, et al (1999) Multilineage potential of adult human mesenchymal stem cells. Science 284: 143–147. 10102814 10.1126/science.284.5411.143

[22] Friedenstein AJ, Chailakhyan RK, Latsinik NV, Panasyuk AF, Keiliss-Borok IV (1974) Stromal cells responsible for transferring the microenvironment of the hemopoietic tissues. Cloning in vitro and retransplantation in vivo. Transplantation 17: 331–340. 4150881 10.1097/00007890-197404000-00001

[23] Tolar J, Le Blanc K, Keating A, Blazar BR (2010) Concise review: hitting the right spot with mesenchymal stromal cells. Stem Cells 28: 1446–1455. 20597105 10.1002/stem.459 PMC3638893

[24] Zuk PA, Zhu M, Mizuno H, Huang J, Futrell JW, et al (2001) Multilineage cells from human adipose tissue: implications for cell-based therapies. Tissue Eng 7: 211–228. 11304456 10.1089/107632701300062859

[25] Erices A, Conget P, Minguell JJ (2000) Mesenchymal progenitor cells in human umbilical cord blood. Br J Haematol 109: 235–242. 10848804 10.1046/j.1365-2141.2000.01986.x

[26] In 't Anker PS, Scherjon SA, Kleijburg-van der Keur C, Noort WA, Claas FH, et al (2003) Amniotic fluid as a novel source of mesenchymal stem cells for therapeutic transplantation. Blood 102: 1548–1549. 12900350 10.1182/blood-2003-04-1291

[27] Gronthos S, Mankani M, Brahim J, Robey PG, Shi S (2000) Postnatal human dental pulp stem cells (DPSCs) in vitro and in vivo. Proc Natl Acad Sci U S A 97: 13625–13630. 11087820 10.1073/pnas.240309797 PMC17626

[28] Kuznetsov SA, Mankani MH, Gronthos S, Satomura K, Bianco P, et al (2001) Circulating skeletal stem cells. J Cell Biol 153: 1133–1140. 11381097 10.1083/jcb.153.5.1133 PMC2174322

[29] Kern S, Eichler H, Stoeve J, Kluter H, Bieback K (2006) Comparative analysis of mesenchymal stem cells from bone marrow, umbilical cord blood, or adipose tissue. Stem Cells 24: 1294–1301. 16410387 10.1634/stemcells.2005-0342

[30] Lv ZD, Wang HB, Dong Q, Kong B, Li JG, et al (2013) Mesothelial cells differentiate into fibroblast-like cells under the scirrhous gastric cancer microenvironment and promote peritoneal carcinomatosis in vitro and in vivo. Mol Cell Biochem 377: 177–185. 23392771 10.1007/s11010-013-1583-0

[31] Lv ZD, Na D, Ma XY, Zhao C, Zhao WJ, et al (2011) Human peritoneal mesothelial cell transformation into myofibroblasts in response to TGF-ss1 in vitro. Int J Mol Med 27: 187–193. 21152863 10.3892/ijmm.2010.574

[32] Tsukada T, Fushida S, Harada S, Yagi Y, Kinoshita J, et al (2012) The role of human peritoneal mesothelial cells in the fibrosis and progression of gastric cancer. Int J Oncol 41: 476–482. 22614335 10.3892/ijo.2012.1490 PMC3582882

[33] Barcellos-Hoff MH, Park C, Wright EG (2005) Radiation and the microenvironment - tumorigenesis and therapy. Nat Rev Cancer 5: 867–875. 16327765 10.1038/nrc1735

[34] Albini A, Sporn MB (2007) The tumour microenvironment as a target for chemoprevention. Nat Rev Cancer 7: 139–147. 17218951 10.1038/nrc2067

[35] Egeblad M, Rasch MG, Weaver VM (2010) Dynamic interplay between the collagen scaffold and tumor evolution. Curr Opin Cell Biol 22: 697–706. 20822891 10.1016/j.ceb.2010.08.015 PMC2948601

[36] Nabha SM, dos Santos EB, Yamamoto HA, Belizi A, Dong Z, et al (2008) Bone marrow stromal cells enhance prostate cancer cell invasion through type I collagen in an MMP-12 dependent manner. Int J Cancer 122: 2482–2490. 18324629 10.1002/ijc.23431 PMC3842601

[37] Koenig A, Mueller C, Hasel C, Adler G, Menke A (2006) Collagen type I induces disruption of E-cadherin-mediated cell-cell contacts and promotes proliferation of pancreatic carcinoma cells. Cancer Res 66: 4662–4671. 16651417 10.1158/0008-5472.CAN-05-2804

[38] Dobbie JW (1992) Pathogenesis of peritoneal fibrosing syndromes (sclerosing peritonitis) in peritoneal dialysis. Perit Dial Int 12: 14–27. 1347465

[39] Uccelli A, Moretta L, Pistoia V (2008) Mesenchymal stem cells in health and disease. Nat Rev Immunol 8: 726–736. 19172693 10.1038/nri2395

[40] Spaggiari GM, Capobianco A, Becchetti S, Mingari MC, Moretta L (2006) Mesenchymal stem cell-natural killer cell interactions: evidence that activated NK cells are capable of killing MSCs, whereas MSCs can inhibit IL-2-induced NK-cell proliferation. Blood 107: 1484–1490. 16239427 10.1182/blood-2005-07-2775

[41] Spaggiari GM, Capobianco A, Abdelrazik H, Becchetti F, Mingari MC, et al (2008) Mesenchymal stem cells inhibit natural killer-cell proliferation, cytotoxicity, and cytokine production: role of indoleamine 2,3-dioxygenase and prostaglandin E2. Blood 111: 1327–1333. 17951526 10.1182/blood-2007-02-074997

[42] Rafii A, Mirshahi P, Poupot M, Faussat AM, Simon A, et al (2008) Oncologic trogocytosis of an original stromal cells induces chemoresistance of ovarian tumours. PLoS One 3: e3894. 19079610 10.1371/journal.pone.0003894 PMC2597737

[43] Martinet L, Poupot R, Mirshahi P, Rafii A, Fournie JJ, et al (2010) Hospicells derived from ovarian cancer stroma inhibit T-cell immune responses. Int J Cancer 126: 2143–2152. 19739080 10.1002/ijc.24881

[44] Pasquet M, Golzio M, Mery E, Rafii A, Benabbou N, et al (2010) Hospicells (ascites-derived stromal cells) promote tumorigenicity and angiogenesis. Int J Cancer 126: 2090–2101. 19739074 10.1002/ijc.24886

[45] Daniels CE, Wilkes MC, Edens M, Kottom TJ, Murphy SJ, et al (2004) Imatinib mesylate inhibits the profibrogenic activity of TGF-beta and prevents bleomycin-mediated lung fibrosis. J Clin Invest 114: 1308–1316. 15520863 10.1172/JCI19603 PMC524221

[46] Abdollahi A, Li M, Ping G, Plathow C, Domhan S, et al (2005) Inhibition of platelet-derived growth factor signaling attenuates pulmonary fibrosis. J Exp Med 201: 925–935. 15781583 10.1084/jem.20041393 PMC2213091

[47] Wang S, Wilkes MC, Leof EB, Hirschberg R (2005) Imatinib mesylate blocks a non-Smad TGF-beta pathway and reduces renal fibrogenesis in vivo. FASEB J 19: 1–11. 15629889 10.1096/fj.04-2370com

[48] Akhmetshina A, Dees C, Pileckyte M, Maurer B, Axmann R, et al (2008) Dual inhibition of c-abl and PDGF receptor signaling by dasatinib and nilotinib for the treatment of dermal fibrosis. FASEB J 22: 2214–2222. 18326784 10.1096/fj.07-105627

[49] Iwamoto N, Distler JH, Distler O (2011) Tyrosine kinase inhibitors in the treatment of systemic sclerosis: from animal models to clinical trials. Curr Rheumatol Rep 13: 21–27. 21042889 10.1007/s11926-010-0142-x

[50] Gordon JK, Spiera RF (2010) Targeting tyrosine kinases: a novel therapeutic strategy for systemic sclerosis. Curr Opin Rheumatol 22: 690–695. 20827202 10.1097/BOR.0b013e32833f1105

[51] Studeny M, Marini FC, Champlin RE, Zompetta C, Fidler IJ, et al (2002) Bone marrow-derived mesenchymal stem cells as vehicles for interferon-beta delivery into tumors. Cancer Res 62: 3603–3608. 12097260

[52] Karnoub AE, Dash AB, Vo AP, Sullivan A, Brooks MW, et al (2007) Mesenchymal stem cells within tumour stroma promote breast cancer metastasis. Nature 449: 557–563. 17914389 10.1038/nature06188

[53] Spaeth EL, Dembinski JL, Sasser AK, Watson K, Klopp A, et al (2009) Mesenchymal stem cell transition to tumor-associated fibroblasts contributes to fibrovascular network expansion and tumor progression. PLoS One 4: e4992. 19352430 10.1371/journal.pone.0004992 PMC2661372

