# Supplementary figures and images for: CD90(+) Mesothelial-Like Cells in Peritoneal Fluid Promote Peritoneal Metastasis by Forming a Tumor Permissive Microenvironment
Source: PLoS One. 2014 Jan 21;9(1):e86516. doi: 10.1371/journal.pone.0086516 (PMC3897715; doi:10.1371/journal.pone.0086516)

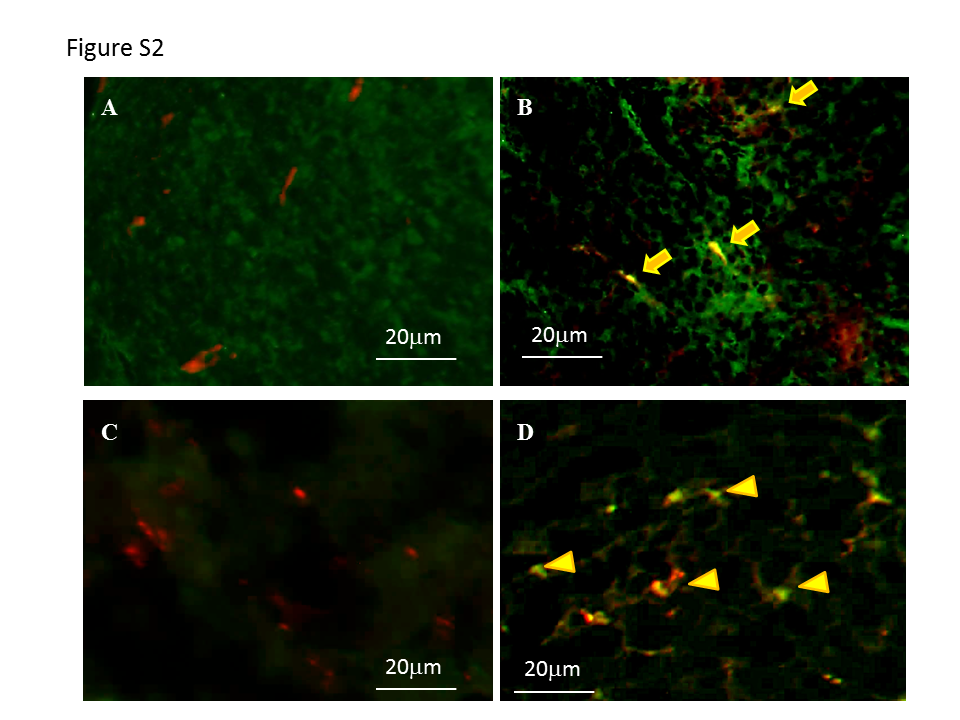

Supplement: Figure S2 — Peritoneal nodules developed in nude mouse after IP injection of MKN45 cells (1×10^6^) and PKH26-labelled MLCs (5×10^5^) were excised, and the tissue sections were immunostained with rabbit antibody to type I collagen (B) or rat antibody to FAP-α (D) followed by the incubation with FITC-conjugated secondary antibody, and observed under fluorescence microscopy. (A) control rabbit IgG, (C) control rat IgG. In merged pictures, PKH26 (+) cells are shown to be positive for Type I collagen (Arrows in B) and for FAP-α (Arrowhead in D). (TIF) [file pone.0086516.s002.tif]

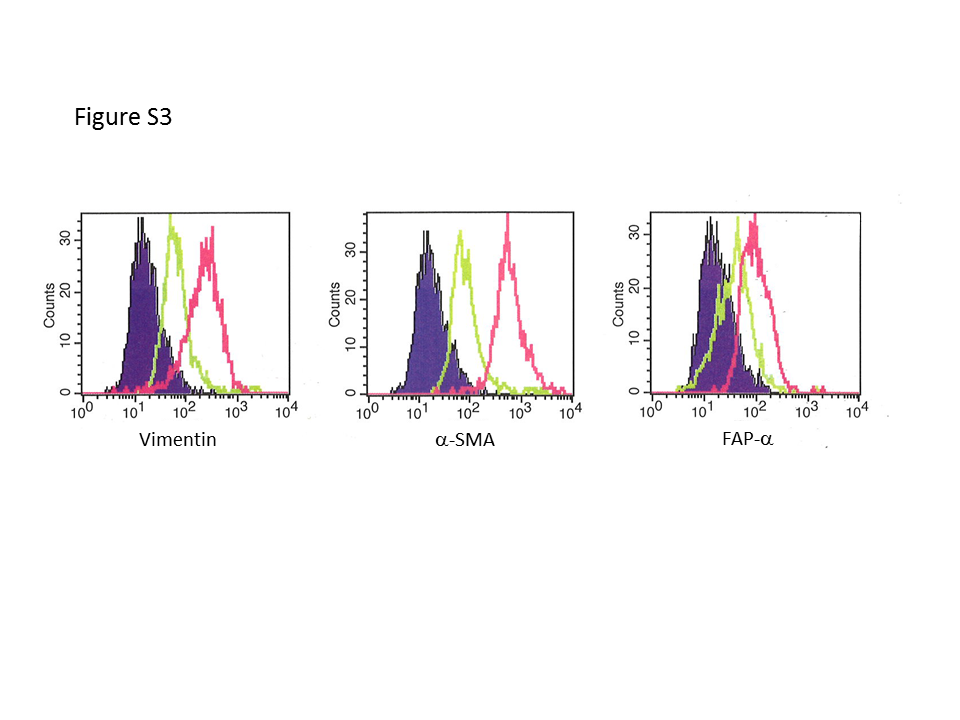

Supplement: Figure S3 — MLC treated with (Red line) or without (Green line) 10 ng/ml TGF-β for 48 hours were detached, fixed, permeabilized and stained with mAbs to Vimentin, α-SMA and FAP-α as described Material and Methods. Shaded profile shows the negative control. (TIF) [file pone.0086516.s003.tif]

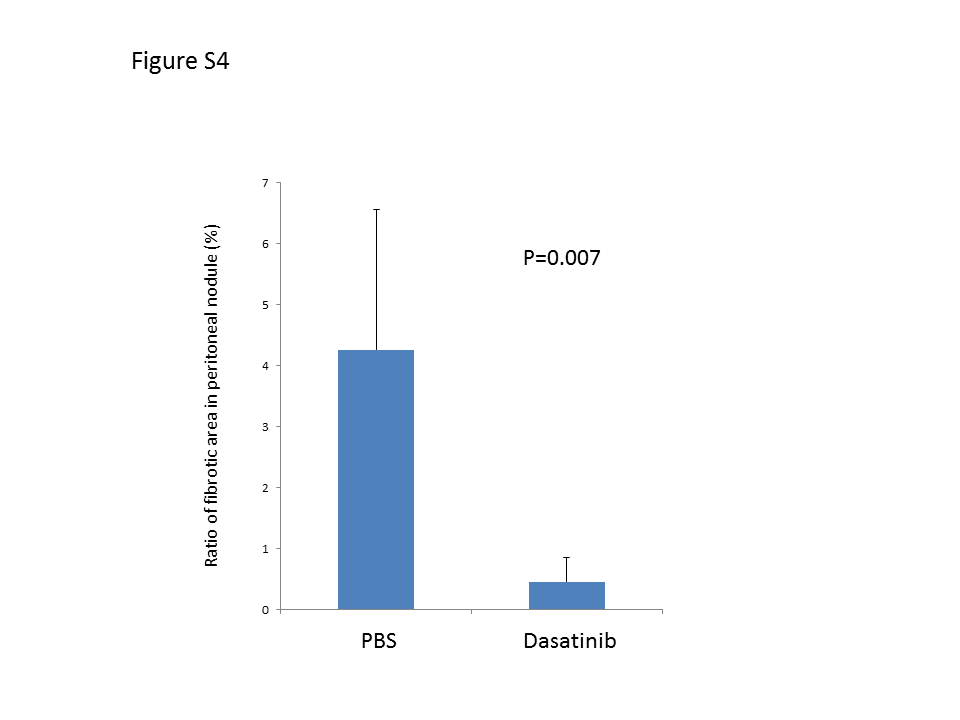

Supplement: Figure S4 — MKN45 cells (1×10^6^) and MLCs (5×10^5^) were co-injected into the peritoneum of nude mice. Dasatinib (50 mg/kg) in 1.0 ml PBS was orally administrated for 14 consecutive days starting 3 days after tumor inoculation. Two weeks later, the mice were sacrificed and macroscopic metastasis in the peritoneum were excised, and tissue sections of peritoneal nodules of control and Dasatinib-treated mice were stained using the Masson-Trichrome method, and the percentages of fibrous area in total area were calculated in randomly selected 10 areas in 5 different tissue sections using a measurement module of BZ-H1M analyzing system (Keyence, Osaka, Japan). (TIF) [file pone.0086516.s004.tif]
